# GT-repeat polymorphism in the heme oxygenase-1 gene promoter and the risk of carotid atherosclerosis related to arsenic exposure

**DOI:** 10.1186/1423-0127-17-70

**Published:** 2010-08-26

**Authors:** Meei-Maan Wu, Hung-Yi Chiou, Te-Chang Lee, Chi-Ling Chen, Ling-I Hsu, Yuan-Hung Wang, Wen-Ling Huang, Yi-Chen Hsieh, Tse-Yen Yang, Cheng-Yeh Lee, Ping-Keung Yip, Chih-Hao Wang, Yu-Mei Hsueh, Chien-Jen Chen

**Affiliations:** 1 School of Public Health, Taipei Medical University, Taipei, Taiwan; 2 Graduate Institute of Oncology, College of Medicine, National Taiwan University, Taipei, Taiwan; 3 Graduate Institute of Basic Medicine, College of Medicine, Fu-Jen Catholic University, Taipei, Taiwan; 4 Institute of Biomedical Sciences, Academia Sinica, Taipei, Taiwan; 5 Graduate Institute of Clinical Medicine, College of Medicine, National Taiwan University, Taipei, Taiwan; 6 Genomics Research Center, Academia Sinica, Taipei, Taiwan; 7 Center of Excellence for Cancer Research, Taipei Medical University, Taipei, Taiwan; 8 School of Medicine, College of Medicine, Fu-Jen Catholic University, Taipei, Taiwan; 9 Department of Cardiology, Cardinal Tien Hospital, College of Medicine, Fu-Jen Catholic University, Taipei, Taiwan

## Abstract

**Background:**

Arsenic is a strong stimulus of heme oxygenase (HO)-1 expression in experimental studies in response to oxidative stress caused by a stimulus. A functional GT-repeat polymorphism in the HO-1 gene promoter was inversely correlated to the development of coronary artery disease in diabetics and development of restenosis following angioplasty in patients. The role of this potential vascular protective factor in carotid atherosclerosis remains unclear. We previously reported a graded association of arsenic exposure in drinking water with an increased risk of carotid atherosclerosis. In this study, we investigated the relationship between HO-1 genetic polymorphism and the risk of atherosclerosis related to arsenic.

**Methods:**

Three-hundred and sixty-seven participants with an indication of carotid atherosclerosis and an additional 420 participants without the indication, which served as the controls, from two arsenic exposure areas in Taiwan, a low arsenic-exposed Lanyang cohort and a high arsenic-exposed LMN cohort, were studied. Carotid atherosclerosis was evaluated using a duplex ultrasonographic assessment of the extracranial carotid arteries. Allelic variants of (GT)n repeats in the 5'-flanking region of the HO-1 gene were identified and grouped into a short (S) allele (< 27 repeats) and long (L) allele (≥ 27 repeats). The association of atherosclerosis and the HO-1 genetic variants was assessed by a logistic regression analysis, adjusted for cardiovascular risk factors.

**Results:**

Analysis results showed that arsenic's effect on carotid atherosclerosis differed between carriers of the class S allele (OR 1.39; 95% CI 0.86-2.25; *p *= 0.181) and non-carriers (OR 2.65; 95% CI 1.03-6.82; *p *= 0.044) in the high-exposure LMN cohort. At arsenic exposure levels exceeding 750 μg/L, difference in OR estimates between class S allele carriers and non-carriers was borderline significant (*p *= 0.051). In contrast, no such results were found in the low-exposure Lanyang cohort.

**Conclusions:**

This exploratory study suggests that at a relatively high level of arsenic exposure, carriers of the short (GT)n allele (< 27 repeats) in the HO-1 gene promoter had a lower probability of developing carotid atherosclerosis than non-carriers of the allele after long-term arsenic exposure via ground water. The short (GT)n repeat in the HO-1 gene promoter may provide protective effects against carotid atherosclerosis in individuals with a high level of arsenic exposure.

## Background

Many of the health hazards caused by arsenic are carcinogenic effects [1,2]. Recently, attention was also paid to the close association of ingested arsenic exposure with the development of cardiovascular disease [3-5]. Epidemiological studies carried out in Taiwan identified several vascular disorders caused by long-term exposure to arsenic in well water. Inorganic arsenic in drinking water is associated with increased risks of cardiovascular mortality, peripheral vascular disease, ischemic heart disease, and cerebral infarction in a dose-response relationship [5]. In a more-recent report, Wang et al. demonstrated a significant biological gradient of long-term arsenic exposure with the prevalence of carotid atherosclerosis [6], further providing evidence of the presence of atherosclerosis induced by arsenic.

Despite the well-documented association between atherosclerotic vascular disease and inorganic arsenic in human populations, only a small percentage of arsenic-exposed individuals develop vascular disorders in their lifetime [7,8]. This implies the existence of modifying factors involved in the disease process that result in a subgroup being susceptible to arsenic-associated cardiovascular disorders. The nutritional status, arsenic metabolite profile, and several genetic susceptibility factors were described [4,9]. Among these, inherited risk factors affecting the pathogenesis of atherosclerosis underlying the cardiovascular disorders caused by arsenic have not been fully examined. Particularly, potential susceptibility genes such as those regulating the adaptive response to arsenic exposure have yet to be characterized.

Atherosclerosis is brought about by continuous oxidative stress to artery walls, thereby leading to the concept that inflammation and endogenous antioxidant pathways may play important roles in the development of atherosclerosis [10]. In the initiation phase of atherosclerosis, oxidant-elicited inflammation causes dysfunction of endothelial cells, while endogenous antioxidants reduce vascular injuries and prevent the development of atherosclerosis [10]. Other oxidant-induced gene products in the adaptive/protective response of vessel walls to oxidative stress were also proposed [11]. One such stress-induced protein that may possibly be involved is heme oxygenase (HO). HO is the rate-limiting enzyme in heme degradation, decomposing heme into free iron, biliverdin, and carbon monoxide (CO). Biliverdin is subsequently converted into bilirubin. Recent studies showed that HO-1, an inducible isoform of HO, can be rapidly upregulated by diverse stimulators associated with various cardiovascular disorders [12]. Biliverdin and bilirubin have the effect of scavenging oxygen radicals and reducing the formation of peroxidation products [13]. CO can down-modulate macrophage inflammation and smooth muscle cell proliferation which reduces vascular events [13].

Induction of HO-1 is primarily controlled at the level of transcription initiation. The 5'-flanking region contains varying lengths of GT repeats 526 bp upstream of the transcription site [14]. The number of GT repeats, (GT)n, was shown to influence the inducibility of the gene promoter under oxidative stimulus; the short polymorphic allele leads to high HO-1 inducibility [15,16]. Length polymorphism of the HO-1 gene promoter is inversely correlated to the development of coronary artery disease in high-risk individuals [15,17,18] and of restenosis after clinical angioplasty [19]. However, there are few studies on the relationship of HO-1 with environmentally related cardiovascular disease or subclinical atherosclerosis. Because HO-1 is an early-response molecule and may provide protection from cell damage, we hypothesized that there is reduced risk of atherosclerotic lesions for those persons that display short (GT)n repeats in the HO-1 gene promoter when exposed to an environmental toxin such as arsenic.

Arsenic is an oxidant producer and a strong stimulus of HO-1 expression in cell cultures as a part of the cellular response to oxidative stress to prevent cell damage [20]. However to date, no human data have justified the observation of HO-1 in cell culture. We previously reported on apparently healthy human subjects in whom transcripts levels of the HO-1 gene increased with arsenic in the blood in a dose-dependent pattern, indicating that HO-1 induction is one of the early responses in arsenic-exposed human beings [21]. The relevance of HO-1 induction to arsenic-associated cardiovascular disorders is not known. The aim of the present study was to test the hypothesis that HO-1 induction has a protective effect against atherosclerosis in arsenic-exposed individuals. We assessed the frequency of HO-1 (GT)n repeat genotypes and examined the relationship between HO-1 gene variability and the risk of atherosclerotic lesions in two cohorts from arsenic-exposure areas in Taiwan.

## Methods

### Study areas and cohorts

This study recruited participants from two endemic areas of arsenic exposure in Taiwan: the Lanyang Basin in the northeastern coastal region and the Blackfoot disease (BFD)-endemic area in the southwestern coastal region [22]. Epidemiological biomarker studies were launched as part of a long-term follow-up study on health hazards as well as to explore risk factors other than arsenic exposure, in 1988 [23,24] and 1997 [25,26], respectively, for the two arseniasis-endemic areas.

The arsenic content in well water in the Lanyang Basin area ranged from undetectable (< 0.15 μg/L) to > 3000 μg/L, with median arsenic concentrations of undetectable to 140 μg/L [27]. Residents in this area used their household-owned well water from the late 1940s until the early 1990s, when a government-sponsored water supply system was implemented. During initial health examinations in 1998-1999, a random sample of 687 cohort members who completed an ultrasonographic assessment of the extracranial carotid artery (ECCA) was studied and reported in previous studies [25,26]. Among them, 530 members (77.1%) gave their consent and provided DNA samples for this research. The study protocol was approved by the Institutional Review Board at Taipei Medical University. This subcohort is hereafter called the Lanyang cohort.

In the BFD-endemic area, we focused on three BFD-hyper-endemic villages, consisting of Homei (L, village designation), Fuhsin (M), and Hsinming (N) in Putai Township [23,24]. Residents in these three villages began using arsenic-tainted artesian (> 300 m) well water in the early 1910s. The arsenic level in the artesian well water ranged 90-1700 μg/L, with a median of 400-874 μg/L. A public water supply system was introduced in the early 1960s, and the artesian well water was no longer used after the mid-1970s. In a follow-up health examination in 1996, an ultrasonographic assessment of ECCA atherosclerosis was conducted for the first time. In total, 436 cohort members completed the ECCA assessment during this examination [6]. Among them, 383 members (87.8%) gave their consent and provided DNA samples for this research. The study protocol was approved by the Institutional Review Board at College of Public Health National Taiwan University. This subcohort is hereafter called the LMN cohort.

### Study subjects, questionnaire data, and biochemical assay

To assess the extent and severity of atherosclerosis, we used a high-resolution duplex ultrasound system with B-mode and Doppler scanners (SONOS 1000, Philips, USA) to examine the ECCA for each participant (Hewlett-Packard Sono 1000, Philips, USA). Duplex scanning and operation as well as the definition of carotid atherosclerosis were described in previous studies [6,26]. Briefly, the presence of carotid atherosclerosis was evaluated based mainly on two parameters: the maximal ECCA intimal-medial thickness (IMT) and the presence of ECCA plaque. The maximal IMT was measured on the far side of the common carotid artery (CCA) at the most stenotic location between 0 and 1 cm proximal to the carotid bifurcation. The presence of ECCA plaque was defined as a wall thickening ≥ 50% of the adjacent IMT and assessed at five carotid artery segments, including the proximal CCA, distal CCA, bulb, internal carotid artery, and external carotid artery. Participants having carotid atherosclerosis were defined as patients according to a maximal ECCA IMT of ≥ 1.0 mm or the presence of observable plaque in any of the five carotid artery segments. The remaining participants with no indications constituted the control group.

Information on demographic and lifestyle characteristics was obtained from the baseline questionnaire and updated through a supplemental questionnaire if necessary. Biochemical variables, including total cholesterol, triglycerides, and glucose level in fasting blood were assayed in the year of the ECCA assessment. All laboratory analyses were performed using a standard automatic analyzer. Height, weight, systolic blood pressure, and diastolic blood pressure were measured according to standard protocols. Hypertension was defined as (1) an average systolic blood pressure of ≥ 140 mmHg, (2) an average diastolic blood pressure of ≥ 90 mmHg, or (3) a history of being diagnosed as hypertensive or having taken antihypertensive medication. Subjects were considered to have diabetes, if they had ever been diagnosed by a physician as being diabetic, or had a fasting blood sugar level of ≥ 126 mg/dL.

### Index for arsenic exposure

To evaluate arsenic exposure in one's lifetime for each study subject, a detailed history of residential addresses and duration of artesian well water use were obtained from a personal interview according to a structured questionnaire. In the Lanyang Basin area, well water samples were collected from each household, and the arsenic content in the well water was determined during 1991-1994, by a method of hydride-generation atomic absorption spectrometry [27]. Since residents of the Lanyang cohort had used their own wells, on a household basis, and had drunk water from those wells for more than 50 years, the arsenic concentration in the well water was used to estimate the arsenic exposure of the Lanyang participants.

On the other hand, residents of the LMN cohort had at one time shared one or several artesian wells because of economic reasons, and some of the LMN participants had even moved from one village to another within the BFD-endemic area. To reflect the overall exposure to ingested arsenic for the LMN participants, a cumulative arsenic exposure from drinking well water was applied to represent the arsenic exposure as usually used in our previous reports [23,24]. The cumulative arsenic exposure was calculated as the sum of the products derived by multiplying the arsenic concentration in the well water by the number of years a participant had been drinking that well water while living in any of the various villages. Median levels of arsenic in the well water of the villages where the study subjects had lived were obtained from a report of previous studies carried out in the 1960s [28]. To be compatible with the Lanyang cohort, an index of average arsenic exposure from consuming well water was presented, which was derived by dividing the cumulative arsenic exposure by the years of consuming artesian well water during the subject's lifetime [29].

For the LMN cohort, a total of 95 participants were excluded due to a lack of information regarding arsenic exposure. The median level of arsenic concentration was unknown for some villages in the BFD-endemic area, in which case, the cumulative arsenic exposure or average arsenic exposure of a study subject was classified as unknown and thus removed from the analysis. The proportion of missing values for arsenic exposure was 24.8%, which is similar to those reported in previous studies [29,30].

### HO-1 (GT)n repeat polymorphism

Genomic DNA was extracted from leukocytes in the buffy coat using the Puregene DNA isolation kit (Gentra System, Minneapolis, MN, USA). The 5'-flanking region containing the (GT)n repeats of the HO-1 gene was amplified by the polymerase chain reaction (PCR) with a FAM-labeled sense primer, 5'-AGAGCCTGCAGCTTCTCAGA-3', and an unlabeled antisense primer, 5'-ACAAAGTCTGGCCATAGGAC-3', according to a published sequence by Kimpara et al. [31]. The sizes of the PCR products were analyzed by the National Genotyping Center of Academia Sinica, Taiwan. In short, the PCR products were mixed with the DNA ladder (35-500-bp range; Applied Biosystems, Foster City, CA, USA) and analyzed on a laser-based automatic DNA sequencer (ABI Prism 377). The respective sizes of the (GT)n repeats for each participant were then calculated using the software, GeneMapper vers. 3.0, ABI Prism.

To adjust for the variation resulting from different batches of gel electrophoresis, we prepared six cloned alleles and included them in every run of the capillary electrophoresis for the sample allele analysis as stated above. The repeat numbers of the cloned alleles as control DNA were 16, 20, 23, 27, 30, and 35 (GT). To confirm the sizes of the (GT)n repeats in the control DNA, their PCR products were subcloned into a pCRII vector (Invitrogen, Foster City, CA, USA), and the purified plasmid DNA was subjected to sequence analysis. Using the allele sizing information obtained from these control DNAs, an adjustment to compensate for the variation in different batches was applied to all sample data. This external adjustment step in genotype binning with capillary electrophoresis increases the precision of allele sizing [32]. As to the genotyping accuracy, 5% of random samples were duplicated in the PCR products sequencing and binning adjustment. The agreement between the samples was 100%.

### Monocyte chemotactic protein (MCP)-1 protein levels

To examine the biological effect of HO-1 gene variants, a random sample of 214 control participants (50% of total controls) was selected for the assay of MCP-1 protein levels in serum. Among them, 16 participants were excluded because of hemolysis disturbance, resulting in a final sample of 198. MCP-1 levels in serum were measured by an enzyme-linked immunosorbent assay (Biotrak, Piscataway, NJ, USA) according to the manufacturers' instructions. The lower limit of detection of the assays was 20.5 pg/mL.

### Statistical analysis

In the Lanyang cohort, HO-1 genotypes of 24 subjects were unsuccessfully assayed. In the LMN cohort, the ECCA reading values of seven study subjects were classified as unknown. We therefore excluded these 31 study subjects, resulting in a total of 281 and 506 subjects in the Lanyang and LMN cohorts, respectively, being used throughout the analysis.

For the statistical analysis, we first used a logistic regression model to identify conventional risk factors in relation to cardiovascular disease while adjusting for age and sex distributions. Factors achieving *p *< 0.1 in the age- and gender-adjusted regression were entered as possible confounding variables in the subsequent analysis of arsenic's effect on carotid atherosclerosis. The effect of a risk factor was expressed as an odds ratio (OR) and a 95% confidence interval (CI). All risk factors in the present study were defined as categorical variables in the regression modeling, unless otherwise indicated. Allele repeats were divided into two classes, short (S) or long (L) based on the distribution reports of previous studies [15,16] and ours of this study.

To evaluate whether there was an interactive effect between the HO-1 length polymorphism and arsenic exposure for the risk of developing atherosclerosis, we first estimated the risk associated with arsenic exposure according to the presence or absence of short (GT)n repeats among the participants (carriers of the S/S or S/L genotype vs. carriers of the LL genotype). In the next combination analysis, the relative percentage change in the risk of atherosclerosis from carriers to non-carriers of the class S allele was also measured by arsenic exposure. All analyses were performed using SAS (Win8e; SAS, Cary, NC, USA) statistical software, and the statistical significance level was defined as *p *< 0.05.

## Results

### Conventional risk factors and carotid atherosclerosis

Table 1 presents the frequency distribution and the age- and gender-adjusted ORs with the 95% CIs for the classic risk factors for the patient and control groups of the two cohorts. Aging and being male gender were the two common risk factors that had the strongest effects on carotid atherosclerosis in the study cohorts. In the Lanyang cohort, having a history of hypertension was significantly associated with an increased risk of carotid atherosclerosis. Although statistically not significant, the frequency of total cholesterol of ≥ 200 mg/dL or triglycerides of ≥ 150 mg/dL was found to be higher in the patient group compared to the control group (0.05 ≤ *p *< 0.10). Other factors, including habitual smoking, body-mass index (BMI), and a history of diabetes, revealed no evidence of being associated with carotid atherosclerosis in the Lanyang cohort. On the other hand, having a history of diabetes was a significant risk factor, and having a history of hypertension was found to be associated with a borderline significance level (0.05 ≤ *p *< 0.10), with an increased risk of carotid atherosclerosis in the patient group of the LMN cohort. These factors associated with carotid atherosclerosis at a significant or borderline level were included in further analyses.

**Table 1:** Conventional risk factors and HO-1 genotype in relation to carotid atherosclerosis.

	Lanyang cohort			LMN cohort		
		
	Controls	Patients	Age- and gender-adjusted	Controls	Patients	Age- and gender-adjusted
Characteristics	n (%)	n (%)	OR (95% CI)	n (%)	n (%)	OR (95% CI)
Total subjects	256	250		164	117	
Age, year						
< 55	70 (27.3)	25 (10.0)	1.0	90 (54.9)	22 (18.8)	1.0
55-65	117 (45.7)	94 (37.6)	2.13 (1.25-3.64)^†^	57 (34.8)	49 (41.9)	3.68 (1.99-6.83)^‡^
≥ 65	69 (27.0)	131 (52.4)	4.92 (2.85-8.50)^‡^	17 (10.4)	46 (39.3)	11.33 (5.39-23.83)^‡^
Gender						
Female	154 (60.2)	116 (46.4)	1.0	91 (55.5)	40 (34.2)	1.0
Male	102 (39.8)	134 (53.6)	1.46 (1.01-2.11)*	73 (44.5)	77 (65.8)	2.47 (1.41-4.32)^†^
Habitual smoking						
No	182 (71.1)	146 (58.4)	1.0	133 (81.0)	79 (68.1)	1.0
Yes	74 (28.9)	104 (41.6)	1.10 (0.61-1.97)	31 (18.9)	37 (31.9)	1.29 (0.62-2.71)
Body mass index, kg/m^2^						
< 27	200 (79.4)	207 (83.8)	1.0	134 (81.7)	100 (85.5)	1.0
≥ 27	52 (20.6)	40 (16.2)	0.84 (0.52-1.35)	30 (18.3)	17 (14.5)	0.79 (0.38-1.67)
Triglycerides, mg/dL						
< 150	197 (77.6)	174 (70.2)	1.0	121 (73.8)	78 (67.2)	1.0
≥ 150	57 (22.4)	74 (29.8)	1.48 (0.97-2.25)	43 (26.2)	38 (32.8)	1.07 (0.59-1.95)
Total cholesterol, mg/dL						
< 200	136 (53.5)	115 (46.2)	1.0	78 (47.6)	40 (34.5)	1.0
≥ 200	118 (46.5)	134 (53.8)	1.43 (0.99-2.08)	86 (52.4)	76 (65.5)	1.44 (0.81-2.57)
Hypertension history						
No	168 (65.9)	134 (54.0)	1.0	105 (64.0)	50 (42.7)	1.0
Yes	87 (34.1)	114 (46.0)	1.58 (1.08-2.31)*	59 (36.0)	67 (57.3)	1.65 (0.95-2.87)
Diabetes mellitus						
No	222 (87.4)	221 (88.8)	1.0	137 (84.1)	82 (71.3)	1.0
Yes	32 (12.6)	28 (11.2)	0.84 (0.48-1.47)	26 (16.0)	33 (28.7)	2.57 (1.31-5.03)^†^
HO-1 Genotype						
L/L	65 (25.4)	72 (28.8)	1.0	45 (27.4)	36 (30.8)	1.0
L/S	129 (50.4)	131 (52.4)	0.88 (0.57-1.35)	90 (54.9)	54 (46.2)	0.49 (0.25-0.96)*
S/S	62 (24.2)	47 (18.8)	0.69 (0.41-1.17)	29 (17.7)	27 (23.1)	0.75 (0.33-1.66)

OR: odds ratio; CI: confidence interval.

Age was defined as continuous variable in the age- and gender-adjusted regression models.

Difference from the total number of patients and controls for each variable is due to missing data.

The class S allele denotes < 27 GT repeats and L allele ≥ 27 GT repeats in the HO-1 gene promoter.

* *p *< 0.05, † *p *< 0.01, ‡ *p *< 0.001.

### HO-1 GT repeat polymorphism and the carotid atherosclerosis index

**Figure 1:**
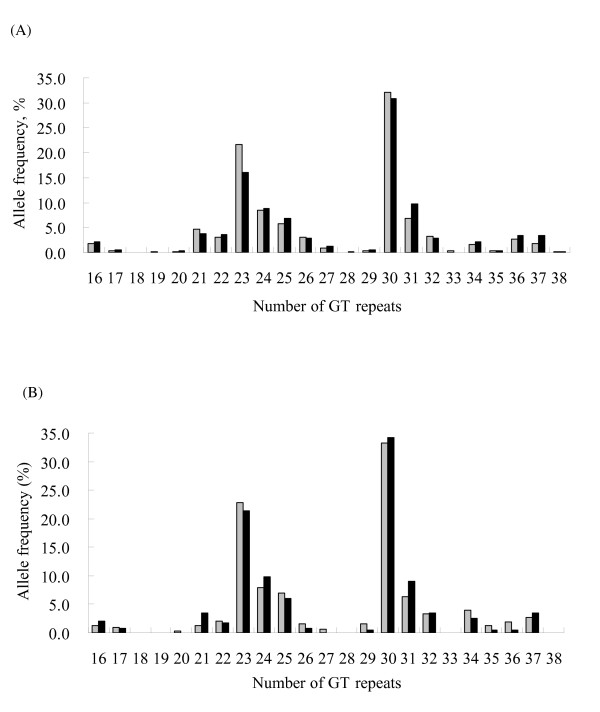
**Frequency distribution of the number of GT repeats in patients having carotid atherosclerosis index (Black) and in controls none the index (Grey) in (A) Lanyang cohort and (B) LMN cohort**.

The number of GT repeats in the HO-1 gene promoter of the participants ranged 16-38 (Figure 1). In both cohorts, 23 and 30 GT repeats were the two most common alleles, which is consistent with findings from previous reports [15,16]. We thus selected 27 GT repeats as a cutoff to classify study subjects in the genetic analysis. (GT)n repeats of < 27 were designated the short (S) allele, and repeats of ≥ 27 as the long (L) allele. Genotype distributions in all groups were in Hardy-Weinberg equilibrium. Analysis results showed that there were no statistically significant differences in the genotype distribution of (GT)n repeats between controls and patients with carotid atherosclerosis (Table 1). Interestingly, we observed a higher frequency of the class S allele or a higher frequency of genotypes containing the class S allele in the control groups compared to the patient groups in both cohorts.

### Association of arsenic exposure with carotid atherosclerosis

As shown in Table 2, the age- and gender-adjusted analysis results demonstrated an increased risk of carotid atherosclerosis with an increase in the arsenic concentration in well water in a dose-response pattern for both study cohorts. Results from the Lanyang cohort indicated a significant association between atherosclerosis and levels of arsenic exposure in well water after taking into account the logarithm of triglycerides, total cholesterol, and hypertension history. For participants in the LMN cohort, the association observed in the prior age- and gender-adjusted analysis remained significant after additional adjustment for a hypertension history and diabetes history.

**Table 2:** Arsenic exposure and carotid atherosclerosis.

Average arsenic exposure, μg/L	Controls n (%)	Patients n (%)	Age- and gender-adjusted OR (95% CI)	Multivariate-adjusted OR (95% CI)
*Lanyang cohort*^*a*^				
≤ 10	18 (7.0)	9 (3.6)	1.00 (referent)	1.00 (referent)
10.1-50	10 (3.9)	9 (3.6)	2.54 (0.71-9.06)	2.58 (0.70-9.56)
50.1-100	87 (34.0)	88 (35.2)	2.74 (1.13-6.64)*	2.98 (1.21-7.34)*
100.1-300	79 (30.9)	81 (32.4)	2.82 (1.16-6.87)*	3.07 (1.23-7.65)*
> 300	62 (24.2)	63 (25.2)	2.49 (1.01-6.15)*	2.62 (1.04-6.60)*
*LMN cohort*^*b*^				
≤ 300	52 (31.7)	12 (10.3)	1.00 (referent)	1.00 (referent)
300-750	65 (39.6)	56 (47.9)	2.03 (0.86-4.77)	1.93 (0.81-4.60)
> 750	47 (28.7)	49 (41.9)	2.70 (1.12-6.47)*	2.78 (1.14-6.78)*
Trend test			1.56 (1.04-2.34)*	1.61 (1.06-2.45)*

OR: odds ratio; CI: confidence interval.

^a ^Adjusted for age, sex, logarithm triglyceride, total cholesterol, and hypertension history.

^b ^Adjusted for age, sex, hypertension history, and diabetes history.

Age, triglyceride, and cholesterol were defined as continuous variables in the regression models.

* *p *< 0.05.

### Interaction between HO-1 (GT) repeat genotypes and arsenic exposure

In the multivariate models including conventional risk factors, the effect of arsenic exposure seemingly differed between carriers of the class S allele and non-carriers of the allele in the LMN cohort according to analysis results of a trend test for arsenic exposure by the HO-1 genotype (Table 3). In carriers of the class S allele, arsenic exposure had a low OR for atherosclerosis indication (OR 1.39; 95% CI 0.86-2.25; *p *= 0.181), whereas in non-carriers, arsenic exposure was associated with a high OR (OR 2.65; 95% CI 1.03-6.82; *p *= 0.044). In contrast, no such result was found in the Lanyang cohort. In a further analysis of the combined effect of arsenic exposure and HO-1 genotype (carriers or non-carriers of the class S allele), no significant OR estimates were found for any subdivided groups in either cohort (Table 4). We noted that the OR estimates were consistently lower in carriers with the class S allele than non-carriers in all comparisons by arsenic exposure group in both cohorts (Table 4). The difference in OR estimates between class S allele carriers and non-carriers reached borderline significance (*p *= 0.051) at an level arsenic exposure exceeding 750 μg/L in the LMN cohort.

**Table 3:** Association of arsenic exposure with carotid atherosclerosis by carriers and non-carriers of the class S allele in the HO-1 gene promoter.

	Carriers of the class S allele			Non-carriers of the class S allele		
		
Arsenic exposure, μg/L	Controls n (%)	Patients n (%)	Multivariate-adjusted OR (95% CI)	Controls n (%)	Patients n (%)	Multivariate-adjusted OR (95% CI)
*Lanyang cohort*^*a*^						
≤ 10	13 (6.8)	7 (3.9)	1.0 (referent)	5 (7.7)	2 (2.8)	1.0 (referent)
10.1-50	7 (3.7)	5 (2.8)	1.57 (0.33-7.38)	3 (4.6)	4 (5.6)	8.64 (0.70-106.82)
50.1-100	66 (34.6)	64 (36.0)	2.90 (1.02-8.27)*	21 (32.3)	24 (33.3)	4.04 (0.64-25.76)
100.1-300	60 (31.4)	61 (34.3)	2.90 (1.00-8.35)*	19 (29.2)	20 (27.8)	4.47 (0.69-29.19)
> 300	45 (23.6)	41 (23.0)	2.39 (0.81-7.05)	17 (26.2)	22 (30.6)	3.97 (0.62-25.57)
Trend test			1.12 (0.91-1.38)			1.13 (0.81-1.57)
*LMN cohort*^*b*^						
≤ 300	39 (32.8)	10 (12.4)	1.0 (referent)	13 (28.9)	2 (5.6)	1.0 (referent)
300-750	43 (36.1)	36 (44.4)	2.04 (0.75-5.57)	22 (48.9)	20 (55.6)	1.13 (0.16-7.95)
> 750	37 (31.1)	35 (43.2)	2.20 (0.79-6.10)	10 (22.2)	14 (38.9)	4.65 (0.66-32.94)
Trend test			1.39 (0.86-2.25)			2.65 (1.03-6.82)*

OR: odds ratio; CI: confidence interval.

^a ^Adjusted for age, sex, logarithm triglyceride, total cholesterol, and hypertension history.

^b ^Adjusted for age, sex, hypertension history, and diabetes history.

Age, triglyceride, and cholesterol were defined as continuous variables in the regression models.

The class S allele denotes <27 GT repeats and L allele ≥27 GT repeats in the HO-1 gene promoter.

* *p *< 0.05.

**Table 4:** Combined effect of arsenic exposure and HO-1 genotype on the risk of carotid atherosclerosis.

Combination of arsenic exposure, μg/L and HO-1 genotype	Controls n (%)	Patients n (%)	Multivariate-adjusted OR (95% CI)	OR changes for carriers vs. non-carriers of the class S allele, %
*Lanyang cohort*^*a*^				
≤ 50, Non-carriers of the class S allele	8 (3.1)	6 (2.4)	1.00	(Reference)
≤ 50, Carriers of the class S allele	20 (7.8)	12 (4.8)	0.60 (0.15-2.45)	-40.0
50-100, Non-carriers of the class S allele	21 (8.2)	24 (9.6)	1.49 (0.39-5.69)	(Reference)
50-100, Carriers of the class S allele	66 (25.8)	64 (25.6)	1.38 (0.40-4.80)	-7.4
100-300, Non-carriers of the class S allele	19 (7.4)	20 (8.0)	1.72 (0.44-6.70)	(Reference)
100-300, Carriers of the class S allele	60 (23.4)	61 (24.4)	1.37 (0.39-4.79)	-20.3
> 300, Non-carriers of the class S allele	17 (6.6)	22 (8.8)	1.48 (0.38-5.77)	(Reference)
> 300, Carriers of the class S allele	45 (17.6)	41 (16.4)	1.14 (0.32-4.08)	-23.0
*LMN cohort*^*b*^				
≤ 300, Non-carriers of the class S allele	13 (7.9)	2 (1.7)	1.00	(Reference)
≤ 300, Carriers of the class S allele	39 (23.8)	10 (8.6)	0.49 (0.07-3.32)	-51.0
300-750, Non-carriers of the class S allele	22 (13.4)	20 (17.1)	1.13 (0.18-7.16)	(Reference)
300-750, Carriers of the class S allele	43 (26.2)	36 (30.8)	1.02 (0.17-6.10)	-9.7
> 750, Non-carriers of the class S allele	10 (6.1)	14 (12.0)	4.45 (0.64-30.93)	(Reference)
> 750, Carriers of the class S allele	37 (22.6)	35 (29.9)	1.09 (0.18-6.64)	-75.5*

OR: odds ratio; CI: confidence interval.

^a ^Adjusted for age, sex, logarithm triglyceride, total cholesterol, and hypertension history.

^b ^Adjusted for age, sex, hypertension history, and diabetes history.

Age, triglyceride, and cholesterol were defined as continuous variables in the regression models.

The class S allele denotes <27 GT repeats and L allele ≥27 GT repeats in the HO-1 gene promoter.

* *p *= 0.051 for the OR difference between carriers vs. non-carriers of the class S allele at arsenic exposure level >750 μg/L.

### Serum MCP-1 levels in carriers versus non-carriers of the class S allele

We also examined the influence of the HO-1 genotype on serum MCP-1 protein levels, an indicator of an inflammatory vessel wall response. We divided the control participants without atherosclerosis indications into three groups, L/L, L/S, and S/S genotypes, and compared serum levels of the MCP-1 protein among the three groups. However, no significant association between HO-1 genotypes and serum MCP-1 levels was found (Additional file 1). Median values with the inter-quartile range were 689 (498-895), 660 (517-833), and 643 (505-842) pg/ml for the L/L, L/S, and S/S genotypes, respectively.

## Discussion

Our previous study demonstrated that oxidative stress levels elevate with an increasing arsenic level in the blood of individuals consuming arsenic-contaminated well water [33]. Among the same study subjects, transcript levels of an inflammation mediator gene and the HO-1 gene increased in dose-response patterns with arsenic exposure [21]. Whether induction of the HO-1 gene in humans is merely a biomarker responding to arsenic exposure without influencing the health or rather an induced response protecting against oxidative damage caused by arsenic remains unknown. This study investigated the relationship between (GT)n repeat polymorphism in the HO-1 gene promoter and the risk of carotid atherosclerosis in arsenic-exposed study cohorts. The cohort members were recruited from two endemic areas that represent, respectively, low- and high-arsenic-exposure areas of Taiwan. In the low-exposure Lanyang cohort, the HO-1 genotype was not significantly associated with carotid atherosclerosis. In the high-exposure LMN cohort, however, our results suggested a borderline significant (*p *= 0.051) lower risk of atherosclerosis indication for carriers of the class S allele (< 27 GT repeats) compared to non-carriers at a high level of arsenic exposure. Analysis results of this study partially support our hypothesis that the short (GT)n repeat allele in the HO-1 gene promoter, which is relevant to high HO-1 induction levels, may protect against atherosclerosis in Taiwanese after long-term high-level arsenic exposure via groundwater.

Our study results did not indicate any particular (GT)n repeat allele in the study participants independently being associated with the risk of carotid atherosclerosis. This finding is consistent with that of previous studies indicating that HO-1 protects against adverse cardiovascular events only in the presence of conventional risk factors or following a clinical intervention [15,17-19]. The above studies indicated no association in the entire sample group without stratification by risk factors [15,18]. In our data, the HO-1 genotype was seemingly associated with atherosclerosis risk only in the subgroup of high-risk individuals with arsenic exposure levels exceeding 750 μg/L. In that particular circumstance, arsenic exposure presumably acted as a strong inducer, and the apparent inability of non-carriers of the class S allele to generate protective proteins against arsenic toxicity may have led to a high risk probability.

HO-1 is a well-known toxicological signature of arsenic treatment in diverse experimental conditions [20]. However, the molecular mechanism by which arsenic induces HO-1 expression has not been clearly defined. Despite the relation of several transcriptional factors to HO-1 induction by arsenic [34], the precise cis-acting elements in the 5'flanking region of the HO-1 gene have not been identified [35,36]. Exactly how arsenic exposure in humans interacts with the (GT)n repeats in the HO-1 gene promoter and how the resulting interaction limits the progression of atherosclerosis need to be elucidated experimentally.

This study also evaluated whether the HO-1 genotype affected systematic levels of inflammation by assaying serum MCP-1 protein levels in study subjects. MCP-1 was evaluated because our previous study involving a group of study subjects recruited from the same cohort demonstrated that arsenic exposure upregulates this inflammatory molecule [21]. Other studies showed that HO-1 induction inhibits MCP-1 expression in cultured cells [37,38]. Therefore, in this study, this marker was used to reflect the anti-inflammatory effect of HO-1 induction in blood vessels after long-term exposure to arsenic. However, analysis results revealed no differential anti-inflammatory response in carriers of the class S allele vs. non-carriers after chronic arsenic exposure. No difference in the inflammatory response between groups could be attributed to the possibility that the MCP-1 expression level is not sufficiently sensitive to determine the differential effect of the HO-1 genotype on arsenic-related atherosclerosis. HO-1 expression is seen throughout the development of atherosclerotic lesions, from early fatty streaks to advanced lesions, in which many cytokines other than MCP-1 are regulated by HO-1 catalytic products during the inflammatory process [11,13]. As for high-level arsenic exposure, the extent to which the HO-1 functional polymorphism affects inflammation molecules in the atherosclerotic process needs to be elucidated.

We recognize that this study has certain limitations. First, a reducing effect from the combination of the HO-1 short (GT)n allele and arsenic exposure, if it exists at all, is only slight and limited to high arsenic exposure; in addition, the statistical testing was of borderline significance. The resulting slight effect could partially be attributed to the many genes involved in atherosclerosis, possibly masking the role of the HO-1 genotype. Gene polymorphisms of p53 and glutathione-transferase P1 (GSTP1) were related to the risk of carotid atherosclerosis in the Lanyang cohort [25]. After adjusting for the influence of the combined p53 and GSTP1 gene polymorphism in the multivariate models, our results indicated no essential change (data not shown). However, we could not exclude the possibility of other genes that confounded the relation between the HO-1 genotype and carotid atherosclerosis. In the LMN cohort in the context of high arsenic exposure, the possibility of an effect from other genes linked to the HO-1 gene or their haplotypic blocks could not be ruled out. Thus, the suggestive borderline association in participants with a high level of arsenic exposure of > 750 μg/L in the LMN cohort might not be attributed to the HO-1 short (GT)n allele, but rather due to linkage disequilibrium with a nearby gene.

Second, this study utilized a cross-sectional design, implying some inherent limitations. For instance, a selection bias cannot be ruled out. This study did not include participants who had died of fatal cardiovascular conditions before the scheduled ECCA examination date. However, unless HO-1 activity has a deleterious effect on late-stage clinical events, the benefits from short (GT)n alleles should not spuriously exist. Moreover, our control groups of both cohorts appeared to be well represented in the allele and genotype distributions, which occurred at a similar frequency to that in previous reports involving East Asians [15,18]. Selection of participants might not have influenced our findings of the relationship between the HO-1 genotype and carotid atherosclerosis in the context of high arsenic exposure. However, our findings are only applicable to survivors of serious cardiovascular events.

Finally, given the wide 95% confidence intervals, the risk estimates may be a chance finding. Therefore, the attenuating effect of the HO-1 short (GT) allele must be interpreted cautiously. Results of this study are exploratory. Future studies with a prospective design are warranted to confirm the above preliminary observations.

## Conclusions

Results of this exploratory study suggest that at a relatively high arsenic exposure level, carriers of the short (GT)n allele (containing < 27 repeats) in the HO-1 gene promoter may have a smaller carotid atherosclerosis risk than non-carriers. To confirm our results, further studies are warranted using a larger sample with improved effect estimates, as well as samples of other arsenic-exposed populations with different ethnic backgrounds. A follow-up study must also be carried out on relationships among the HO-1 length polymorphism, long-term arsenic exposure, and adverse cardiovascular events.

## Competing interests

The authors declare that they have no competing interests.

## Authors' contributions

Study concept and design: MMW, TCL, HYC. Data analysis and interpretation: MMW, CLC, LIH. Drafting the manuscript: MMW. HO-1 genotyping data acquisition: MMW, WLH. ECCA clinical data acquisition: PKY, CHW. MCP-1 assay data acquisition: MMW, TYY, CYL. Funding acquisition: MMW. Cohort database management: LIH, YHW, YCH. Cohort material support: CJC, YMH, HYC.

## Supplementary Material

Additional file 1 **Supplemental figure**. Supplemental figure Click here for file
