# Supplementary material for: GT-repeat polymorphism in the heme oxygenase-1 gene promoter and the risk of carotid atherosclerosis related to arsenic exposure
Source: J Biomed Sci. 2010 Aug 26;17(1):70. doi: 10.1186/1423-0127-17-70 (PMC2939596; doi:10.1186/1423-0127-17-70)
Supplement: Additional file 1 — Supplemental figure. Supplemental figure [file 1423-0127-17-70-S1.DOC]

Supplemental Figure

Box plot describes the HO-1 genotype in relation to the serum MCP-1 levels in 198 control participants with no indication of carotid atherosclerosis. The median and the inter-quartile range (from the 25^th^ to 75^th^ percentile) values are 643 (505-842), 660 (517-833), and 689 (498-895) pg/ml for the S/S, L/S, and L/L genotype, respectively.
